# Methods for handling missing data in serially sampled sputum specimens for mycobacterial culture conversion calculation

**DOI:** 10.1186/s12874-022-01782-8

**Published:** 2022-11-19

**Authors:** Samantha Malatesta, Isabelle R. Weir, Sarah E. Weber, Tara C. Bouton, Tara Carney, Danie Theron, Bronwyn Myers, C. Robert Horsburgh, Robin M. Warren, Karen R. Jacobson, Laura F. White

**Affiliations:** 1 Department of Biostatistics, Boston University School of Public Health, 801 Massachusetts Ave, 3rd Floor, Boston, MA 02119 USA; 2 Center for Biostatistics in AIDS Research in the Department of Biostatistics, Harvard T.H. Chan School of Public Health, Boston, MA USA; 3 Section of Infectious Diseases, Boston Medical Center, Boston, MA USA; 4 Section of Infectious Diseases, Department of Medicine, Boston University School of Medicine, Boston, MA USA; 5 Alcohol, Tobacco and Other Drug Research Unit, South African Medical Research Council, Tygerberg, South Africa; 6 Department of Psychiatry and Mental Health, University of Cape Town, Groote Schuur Hospital, Observatory, Cape Town, South Africa; 7 Brewelskloof Hospital, Worcester, South Africa; 8 Curtin enAble Institute, Faculty of Health Sciences, Curtin University, Perth, Australia; 9 Departments of Epidemiology and Global Health, Boston University School of Public Health, Boston, MA USA; 10 DSI-NRF Centre of Excellence for Biomedical Tuberculosis Research and South African Medical Research Council Centre for Tuberculosis Research, Cape Town, South Africa; 11 Division of Molecular Biology and Human Genetics, Faculty of Medicine and Health Sciences, Stellenbosch University, Cape Town, South Africa

**Keywords:** Longitudinal missing data, Multiple imputation, Survival analysis, Tuberculosis, Culture conversion

## Abstract

**Background:**

The occurrence and timing of mycobacterial culture conversion is used as a proxy for tuberculosis treatment response. When researchers serially sample sputum during tuberculosis studies, contamination or missed visits leads to missing data points. Traditionally, this is managed by ignoring missing data or simple carry-forward techniques. Statistically advanced multiple imputation methods potentially decrease bias and retain sample size and statistical power.

**Methods:**

We analyzed data from 261 participants who provided weekly sputa for the first 12 weeks of tuberculosis treatment. We compared methods for handling missing data points in a longitudinal study with a time-to-event outcome. Our primary outcome was time to culture conversion, defined as two consecutive weeks with no *Mycobacterium tuberculosis* growth. Methods used to address missing data included: 1) available case analysis, 2) last observation carried forward, and 3) multiple imputation by fully conditional specification. For each method, we calculated the proportion culture converted and used survival analysis to estimate Kaplan-Meier curves, hazard ratios, and restricted mean survival times. We compared methods based on point estimates, confidence intervals, and conclusions to specific research questions.

**Results:**

The three missing data methods lead to differences in the number of participants achieving conversion; 78 (32.8%) participants converted with available case analysis, 154 (64.7%) converted with last observation carried forward, and 184 (77.1%) converted with multiple imputation. Multiple imputation resulted in smaller point estimates than simple approaches with narrower confidence intervals. The adjusted hazard ratio for smear negative participants was 3.4 (95% CI 2.3, 5.1) using multiple imputation compared to 5.2 (95% CI 3.1, 8.7) using last observation carried forward and 5.0 (95% CI 2.4, 10.6) using available case analysis.

**Conclusion:**

We showed that accounting for missing sputum data through multiple imputation, a statistically valid approach under certain conditions, can lead to different conclusions than naïve methods. Careful consideration for how to handle missing data must be taken and be pre-specified prior to analysis. We used data from a TB study to demonstrate these concepts, however, the methods we described are broadly applicable to longitudinal missing data. We provide valuable statistical guidance and code for researchers to appropriately handle missing data in longitudinal studies.

**Supplementary Information:**

The online version contains supplementary material available at 10.1186/s12874-022-01782-8.

## Background

Standard treatment for drug-susceptible tuberculosis (TB) disease is a 6-month drug regimen [1]. Following the initiation of therapy, sterilization of sputum is expected to occur within two to 3 months. Clinical, radiological, and bacteriological measures are assessed during early treatment as potential predictors of long-term treatment response [2, 3]. Culture conversion, commonly defined as two consecutive sputum cultures without *Mycobacterium (M.) tuberculosis* present, is the best current surrogate endpoint for long-term treatment success with conversion by 2 months following therapy initiation most predictive of durable cure [4–6]. A common primary endpoint in phase II TB clinical trials of new treatments and longitudinal observational studies is time to culture conversion (TCC), defined as the time from treatment initiation to the first of two consecutive negative cultures. TCC is estimated to assess individuals’ response to treatment and compare the efficacy of treatments.

To calculate TCC, sputum specimens are serially collected for culture at regular intervals. As with all longitudinal studies, missing data can occur. Reasons for missing specimens include missed visits, participant attrition, inability to produce sputum, poor quality specimens, or sample contamination. For these analyses, missing data is especially consequential because the outcome of interest depends on consecutive culture results. Incomplete data can potentially bias TCC estimation. TCC calculation will be differentially affected depending on whether the missed culture result would have been positive or negative had it been observed. An example of theoretical culture data for 12 weeks of treatment and possible patterns of missing samples are shown in Table 1. Individuals may be misclassified as non-converters or the estimated conversion week may be later than the true conversion week had all culture results been observed. This can lead to underestimating the true proportion of culture converters by a certain time point or artificially inflating TCC.

**Table 1:** Hypothetical example of missing data patterns and influence on estimated conversion week

Treatment week												
	1	2	3	4	5	6	7	8	9	10	11	12
Complete	**+**	**+**	**+**	**–**	**+**	**+**	**+**	-*	**–**	**–**	**–**	**–**
Pattern 1^a^	**+**	**+**	**X**	**–**	**+**	**+**	**X**	**X**	**X**	-*	**–**	**–**
Pattern 2^b^	**+**	**+**	**+**	**–**	**+**	**+**	**+**	**–**	**X**	**X**	**X**	**X**
Pattern 3^c^	**+**	**+**	**+**	**–**	**+**	**+**	**+**	**X**	**X**	**X**	**X**	**X**
Pattern 4^d^	**+**	**+**	**+**	**–**	**+**	**+**	**+**	-*	**–**	**–**	**X**	**X**

+=positive culture, − = negative culture, X = culture result missing, *=conversion week

^a^ Culture conversion estimate is delayed to week 10 from missing cultures for weeks 7–9

^b^ Culture conversion is not achieved with last observed culture as negative

^c^ Culture conversion is not achieved with last observed culture as positive

^d^ Missing cultures occur after conversion and do not affect estimated conversion week

^e^ Conversion week defined as first of two consecutive cultures without *Mycobacterium tuberculosis* present

Given our interest in a time to event outcome, a natural way to estimate TCC is through survival analysis. This approach avoids excluding individuals with missing data by censoring. Handling missingness prior to censoring is critical for valid TCC estimation but there is no standard method for managing missing data. The simplest option is available case analysis (ACA) where participants are censored at the time of their first missing sample. Another common approach is to implement “last observation carried forward” (LOCF), where last available culture result is carried forward for all subsequent missing values [7, 8]. Although often used, ACA and LOCF methods are not grounded in statistical theory and are known to be biased [9, 10]. Multiple imputation leverages all available information in decisions on filling in missingness. The result is a complete dataset upon which valid statistical inference and point estimation can be performed [11]. The statistical properties and validity of multiple imputation have been shown to be superior to other simple and naïve methods for addressing missing outcome and covariate data. While multiple imputation is based in statistical theory and generally seen as superior to other ad hoc approaches, in practice it still is not widely adopted [12, 13]. There are other alternative methods for addressing missing data in longitudinal studies that are often used in practice and are valid under certain conditions [13–15]. We choose to focus on multiple imputation as a comparison to ACA and LOCF as it is becoming more popular, is well documented with tutorials in multiple software packages, and is a valuable tool for practitioners.

In this study, we describe three methods for handling missing outcome data: ACA, LOCF, and multiple imputation. We detail the general implementation of each method in the context of a planned survival analysis for TCC using serial weekly sputum collections in a longitudinal TB study. We then apply each method to data from a prospective TB cohort of 261 participants from the Tuberculosis Treatment and Alcohol Use Study (TRUST). Our goal is not to compare methods based on statistical performance, but to provide guidance on the practical implementation of multiple imputation in longitudinal studies with a time-to-event outcome. Treating multiple imputation as the gold standard, we compare methods focusing on the underlying assumptions, survival measure estimates, and conclusions to specific research questions. We provide R code for reproducibility and implementation on GitHub.

## Methods

We describe three methods for handling missing outcome data in longitudinal studies with a time-to-event outcome where the event of interest depends on repeated sampling at consecutive time points. We focus on the implementation of these methods within a survival analysis framework.

### Patterns of missing data

To appropriately handle missing data, it is important to first understand the ways in which missing data can occur and the extent this influences estimation. The impact missing data has on an analysis depends on multiple factors including the proportion of missing data, planned statistical analysis, and the missing data mechanism. There are three missing data mechanisms as defined by Rubin: 1) Missing completely at random (MCAR), 2) Missing at random (MAR), and 3) Missing not at random (MNAR). Data are MCAR if missingness does not depend on the values of the data, both missing or observed. Data are MAR if missingness depends only on the observed values of the data. Data are MNAR if missingness depends on unmeasured variables [16]. For data MCAR or MAR, methods exist for addressing missing data. For data MNAR, applying existing methods can lead to invalid statistical inference and biased estimation. It is important to note that while methods for addressing missing data rely on knowing the missing data mechanism, there exists no formal test to verify the assumption that data are MAR or MCAR.

### Available case analysis (ACA)

ACA uses all available information for each case without modification [16]. When operating in a survival analysis framework where the event depends on samples at consecutive time points, as with culture conversion, we can censor individuals with missing outcome data and avoid excluding them entirely. Individuals are censored at the first time point with a missing culture sample that may influence their TCC calculation. This depends entirely on the definition of culture conversion being used. In this example, since we are using two consecutive negative cultures as the definition of conversion, any occurrence of the following culture sequences would require censoring and are illustrated in Table 1 as patterns 1, 2, and 3 respectively: (positive, missing, missing), (positive, negative, missing), (positive, missing, negative).

ACA is the simplest approach to handling missing data, but is rarely appropriate to use because it relies on the assumption data are MCAR and no formal testing can be performed to assess whether this assumption holds. Implementing ACA when this assumption is clearly violated will lead to biased estimation and a loss of precision [17]. Even in the limited situations where ACA may be acceptable, it is not optimal because a loss in statistical power will still occur.

### Last observation carried forward (LOCF)

LOCF is a single imputation method that is implemented by bringing forward the result from the previous time point for each occurrence of a missing sample. This method is often rationalized as we expect two consecutive measurements in time to be strongly correlated. Historically, LOCF has been used in clinical trials to impute missing values due to participant drop out. In the case of missing culture data, individuals miss sample collection intermittently in addition to being lost to follow up (lost completely). Variations of LOCF have been used where the number of weeks carried forward for consecutive missing values can differ. For this example, we carry forward a single culture result each time there is a missing instance. When there are consecutive missing samples, only the first is imputed and the rest are still considered missing. This results in potentially still having missing data after imputation which is subsequently handled with censoring as described with ACA. To illustrate, if LOCF was implemented for the data in pattern 1 of Table 1, weeks 3 and 7 would be imputed as culture positive and we would censor at week 7 as this is the last present sample before two consecutive missing.

LOCF has been used to impute missing data in TB clinical trials and longitudinal observational studies but there is strong evidence that LOCF can lead to biased results [9, 18]. We describe the most conservative approach to implementing LOCF by carrying forward only 1 week when consecutive missing samples occur. We still assume that two consecutive samples have identical culture results and do not allow for uncertainty when imputing or consider other factors known to be predictive of culture results.

### Multiple imputation

Multiple imputation is a statistical method for analyzing incomplete data that leverages all available information in a data set and results in valid statistical estimation and inference under certain conditions. The validity of MI depends most importantly on the assumption that data are MAR, in addition to the amount of missing data and the quantity of information available being sufficient for imputation. When working in a survival analysis framework, the assumption of non-informative censoring must also be considered. We describe in detail multiple imputation (MI) by fully conditional specification because it is straightforward to implement and is flexible in the types of data it can handle. Both predictors and the outcome of interest can have missing data present.

When imputing repeated measures data, a multi-level imputation is the most theoretically sound approach to take [19]. Treating all observations across time as independent without accounting for the within subject correlation will lead to unaccounted variability in analysis and less conservative results [20, 21]. In this tutorial, we do not describe the process of multi-level imputation as it introduces significant modelling complexity which would diverge from our goal of introducing practitioners to the idea of multiple imputation. Instead, we proceed with a single-level imputation and acknowledge this limitation.

MI imputes missing data on a variable-by-variable basis. An imputation model is specified for each variable with missing observations and then imputation is carried out iteratively. If we have n participants represented in the data with p data elements collected, we can organize this into a matrix of size nxp. For a nxp matrix of data *Y*, let *Y*_*j*_ be the jth column in *Y*, and *Y*_−*j*_ indicates all columns in *Y* except *Y*_*j*_. MI specifies the multivariate distribution *P*(*Y*, *X*, *R*| *θ*) through a set of conditional densities *P*(*Y*_*j*_| *X*, *Y*_−*j*_, *R*, *θ*_*j*_) where *X* is a set of predictors used to impute *Y*_*j*_ and *R* indicates the missing observations of each *Y*_*j*_. This conditional density is used to impute the missing values of each *Y*_*j*_ [22]. Imputation using MI starts by randomly filling in missing values, then iterating over the conditionally specified imputation models. One cycle through all variables is considered one iteration and multiple iterations, usually 5–10, are executed resulting in a single imputed data set. This process is done m times, generating m imputed data sets. There is no strict guidance on the number of data sets to impute, but 20 is standard. There are several algorithms to implement imputation using conditionally specified models. We use the Multiple Imputation by Chained Equations (MICE) algorithm as it is extensively documented and is easily implemented using the MICE package in R [23].

Prior to imputing, an imputation model for each *Y*_*j*_ with missing data must be specified. For MI to be valid, data are assumed to be MAR. All variables predictive of missingness should be included as predictors in the imputation model. Additionally, all variables used in the planned analysis should be included to preserve relationships among variables [19]. In the case of imputing repeated measures data, the problem of overfitting or model non-convergence can occur when using all time points to predict missing values [24]. Alternatively, only time points adjacent to each time point can be included as predictors. Distributional assumptions of *Y*_*j*_ must also be specified. MI is flexible as there is a range of parametric distributions to choose from, such as logistic, Poisson, and normal. Correct model specification is not a simple task as issues with collinearity, overfitting and model non-convergence can occur. Others have detailed common difficulties with model specification and best practices [25].

Following imputation, estimation is conducted separately on each of the m imputed data sets using the desired statistical method for analysis. Estimates and standard errors are then pooled (combined) using Rubin’s rules into an overall estimate and variance–covariance matrix [26]. These rules are based in asymptotic theory and assume inference around the parameter of interest is based on the normal approximation such that $Q-\hat{Q}\sim N\left(0,U\right)$, where *Q* is defined as the true value of the quantity being estimated and $\hat{Q}$ is the estimator obtained through imputation and, in our case, survival analysis modeling. Rubin defined the overall estimate $\hat{Q}$, as the average of ${\hat{Q}}_i,i=1,\dots, m,$ estimates where $\hat{Q}=\frac{1}{m}\sum_{i=1}^m{\hat{Q}}_i$.

The pooled variance is defined as $\overline{U}=\frac{1}{m}\sum_{i=1}^m{U}_i$, where *U*_*i*_ is the variance of ${\hat{Q}}_i$, and the between-imputation variance is $B=\frac{1}{m-1}\sum_{i=1}^m{\left({\hat{Q}}_i-\overline{Q}\right)}^2$. To account for both within-imputation variability and between-imputation variability the total variance of $\hat{Q},$ is defined as: $T=\overline{U}+\left(1+\frac{1}{m}\right)B$. Pooled estimates and variances can then be used to perform standard statistical inference. The result is model output that appears the same as all standard output from statistical software.

We are interested in a time to event outcome and focus on implementing Rubin’s rules with survival analysis estimates, including hazard ratios, survival probabilities, and restricted mean survival time difference (RMSTD). Hazard ratios and survival probabilities are standard measures of effect for time to event outcomes while RMSTD is less common. RMSTD is a measure of the difference in average survival time between a treatment and control group and is equivalent to the difference between the area under the two survival curves [16]. For hazard ratios and survival probabilities, the normality assumption does not hold so we transform estimates prior to pooling. We then apply Rubin’s rules as described above and back transform to the original scale. A complementary log-log transformation for survival probabilities and a log transformation for hazard ratios is standard [20, 21].

### Application

We analyzed data from TRUST, a prospective cohort study conducted in Worcester, Western Cape Province, South Africa [31]. Serial sputum specimens were collected at treatment initiation and weekly for the 11 weeks following. Treatment initiation (Week 0) samples were collected within 3 days of initiating TB therapy. For this analysis, we assumed samples were collected on the same day for each week of follow-up, however, this was not always the case, and in practice, should be considered in analysis. Following decontamination, neutralization and centrifugation the sputum sediment was resuspended in 2 ml phosphate buffered saline. Mycobacterial Growth Indicator Tubes (MGIT) were inoculated with a 500 μl aliquot of the resuspended sediment and incubated at 37 °C in the MGIT 960 instrument (Becton Dickinson). A growth index of 100 was used to indicate a positive culture while a negative culture was defined if the growth index failed to reach 100 after 42 days. *M. tuberculosis* growth was confirmed by Ziehl Neelsen staining and the detection of antigen 85 (Capilia TB Neo Assay; Tauns (Japan)), while contamination was indicated by a negative antigen 85 test and/or growth on blood agar. Misclassification of samples was unlikely as MGIT culture is the gold standard for detecting *M. tuberculosis* with high sensitivity [32].

All participants included in the analysis were positive on Xpert MTB/RIF (Cepheid Sunnyvale, CA) or Xpert MTB/RIF Ultra (Cepheid Sunnyvale, CA) or culture positive with rifampicin susceptible pulmonary TB. The outcome of interest was TCC defined as the first of two consecutive weeks with sputum cultures with no growth of *M. tuberculosis*. We investigated missing data patterns to assess overall missingness and how completeness varied during follow-up. We applied the three methods for handling missing data described above. For each method we examined the association between smear status (concentrated smear), defined as positive if acid-fast bacilli were present on smear microscopy at week 1 and negative otherwise, and culture conversion adjusting for confounders using survival analysis.

We classified samples as missing if there was no collection due to missed visits, poor quality of collected samples, or contamination of collected samples without *M. tuberculosis* present. We also considered samples missing due to a participant’s inability to produce sputum as negative for *M. tuberculosis*. We explored missing data patterns visually and summarized using basic descriptive statistics. We divided culture data for each participant into three visit windows (week 0 to week 3, week 4 to week 7, week 8 to week 11), and categorized each window as complete I if all samples were observed, intermittent (I) if one to three samples were observed or missing (M) if no samples were observed. In addition, we summarized basic descriptive statistics by frequency of missing samples to assess whether there were strong associations between key demographics and missingness that may indicate data MNAR.

We implemented three methods for handling missing data. For ACA, the data was not modified prior to analysis. For LOCF, the previous week’s result was carried forward for each occurrence of a missing sample. If there were multiple consecutive samples missing, we only carried forward one sample and all subsequent samples were left unchanged. We implemented MI to impute any occurrence of a missing culture result, as well as any missing covariates.

For MI to be valid, we assumed our data to be MAR. While this assumption cannot be formally tested, we found no systematic patterns of missingness to indicate data were MNAR. We included all variables we planned to use in the main analyses as well as auxiliary variables in our imputation model. We used the following variables when imputing a given week’s culture result: culture result from previous week, smear result from previous week, age, biologic sex, body mass index (BMI), HIV status, tobacco use, smoked drug use, cavitary disease, and excessive alcohol use. Smoked drug use was defined as self-reported or biologically verified methamphetamine, methaqualone, and/or cannabis use. Excessive alcohol use was defined as an Alcohol Use Disorders Identification Test score ≥ 8 and/or a phosphatidylethanol result ≥50 ng/mL. Tobacco use was defined as any self-reported use. Cavitary disease was scored as presence or absence of lung cavitation on chest x-ray. We used logistic regression to impute culture results and binary covariates and multinomial logit regression to impute variables with more than two categories. As missingness of independent variables was minimal in this data set, they were imputed along with the dependent variable (see Additional File 1). Following convention, we generated 20 imputed data sets. After imputing, analyses were run separately on each imputed data set and estimates were combined using Rubin’s rules. All inference was made using pooled estimates and standard errors [29].

We analyzed our data using survival analysis methods and compared results for the three methods of handling missing data. We fit cox proportional hazards models to examine the association between week 0 smear status and TCC, adjusting for age, biologic sex, HIV, and cavitary disease. We tested the proportional hazards assumption using Shoenfeld residuals. We estimated RMSTD for a time horizon of 8 weeks to compare average times to culture conversion for smear positive versus smear negative participants over 8 weeks of treatment. We estimated unadjusted RMSTD estimates using the area between the two Kaplan-Meier curves and adjusted RMSTD using the ANCOVA-type method of Tian et al. [33].

## Results

The TRUST study enrolled 261 participants between May 1, 2017 and June 1, 2021. We excluded 21 participants due to week 1 cultures without *M. tuberculosis* growth and 2 participants with missing week 0 cultures, leaving 238 participants for this analysis. We analyzed 3132 sputum specimens collected over 12 weeks of follow-up from 238 participants; 210 (88.2%) participants were missing at least one sputum specimen. The median (IQR) number of missing samples per individual was 2 [1, 5]. Samples were most commonly missing due to specimen contamination (440, 14.1%), participants being lost to follow up (239, 7.6%), or intermittently missing weekly sputum collection (197, 6.3%).

Missing data patterns were examined and participants were categorized based on the status of their three visit windows (Table 2). The three most frequent patterns were (C, I, I), (C, I, I), and (I, I, I) with frequencies 58 (24.4%), 42 (17.6%), and 31 (13.0%) respectively. Conversion was highest for patterns (C, I, I) and (C, C, I). For participants missing all samples for weeks 4–7 or weeks 8–11, 5 (3.5%) achieved conversion. Comparing key demographics by frequency of missing samples showed participants were more likely to be missing more than 1 sample if they were living with HIV or had cavitation (Table 3).

**Table 2:** Patterns of missing culture results for three-time windows following treatment initiation

Time Window			Total	Conversion^a^
Weeks 0–3	Weeks 4–7	Weeks 8–11	*N =* 238	*N =* 141
			N(%)	
C	C	C	28 (11.8)	20 (14.3)
C	C	I	42 (17.6)	28 (20)
C	C	M	3 (1.3)	1 (0.7)
C	I	C	17 (7.1)	12 (8.6)
C	I	I	58 (24.4)	33 (23.6)
C	I	M	6 (2.5)	1 (0.7)
C	M	C	1 (0.4)	1 (0.7)
C	M	I	1 (0.4)	0 (0)
C	M	M	1 (0.4)	0 (0)
I	C	C	9 (3.8)	6 (4.3)
I	C	I	14 (5.9)	13 (9.3)
I	C	M	1 (0.4)	1 (0.7)
I	I	C	4 (1.7)	3 (2.1)
I	I	I	31 (13)	20 (14.3)
I	I	M	8 (3.4)	2 (1.4)
I	M	I	1 (0.4)	0 (0)
I	M	M	13 (5.5)	0 (0)

*C* No samples missing, I 1–3 samples missing, M All samples missing

^a^ Conversion achieved if two consecutive cultures without *Mycobacterium tuberculosis* occurs

**Table 3:** Descriptive summmary for basic demographics by frequency of missing sputum samples

	0–1 Samples *N =* 68	2–3 Samples *N* = 43	4–5 Samples *N* = 73	> 5 Samples *N =* 54
Male Sex	46 (67.6%)	28 (65.1%)	44 (60.3%)	28 (51.9%)
Age, Median (Q1, Q3)	40 (31, 49)	36 (28, 50)	36 (26, 49)	38 (25, 46)
BMI				
Underweight	41 (60.3%)	31 (72.1%)	44 (60.3%)	34 (63.0%)
Normal Weight	22 (32.4%)	10 (23.3%)	27 (37.0%)	15 (27.8%)
Overweight and Obese	5 (7.4%)	2 (4.7%)	2 (2.7%)	5 (9.3%)
HIV Positive	10 (14.7%)	17 (40.5%)	22 (30.1%)	21 (38.9%)
Excessive Alcohol Use	43 (63.2%)	25 (58.1%)	47 (64.4%)	35 (64.8%)
Tobacco Use	50 (73.5%)	24 (55.8%)	53 (72.6%)	35 (64.8%)
Cavitation	11 (17.5%)	20 (48.8%)	20 (29.0%)	21 (42.0%)
Smoked substance use	32 (47.1%)	28 (65.1%)	44 (60.3%)	30 (55.6%)

**Fig. 1:**
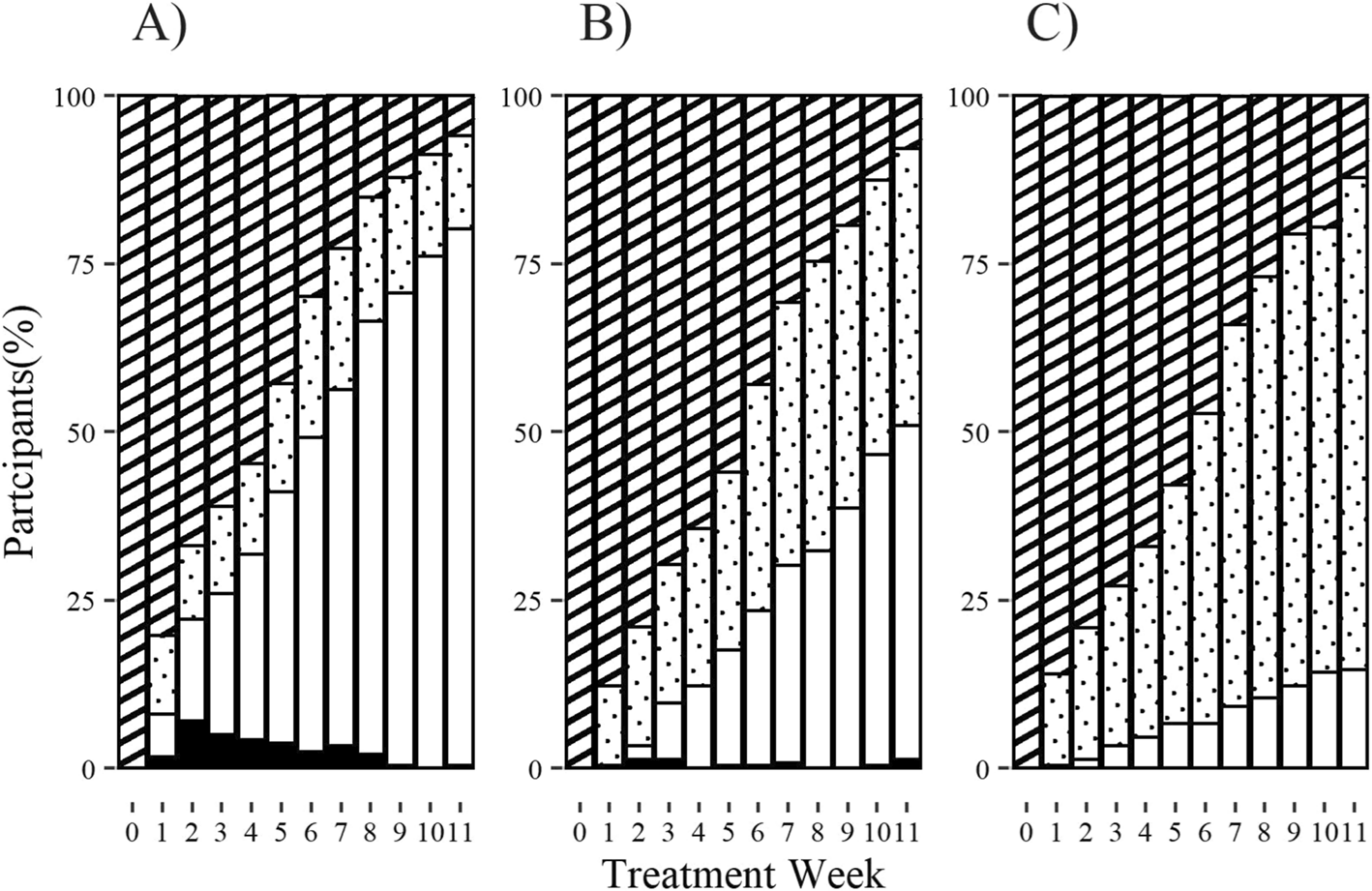
Proportion of participants with positive, negative, or missing cultures per treatment week. Positive and negative culture defined as presence or absence of *Mycobacterium tuberculosis*. Method for handling missing data: available case analysis **A**), last observation carried forward **B**), multiple imputation by fully conditional specification **C**). Stripe, positive; circle, negative; white, censored; black, missing

Following the implementation of each method for handling missing data, the proportion of positive, negative, censored or missing samples each week was calculated (Fig. 1). Compared to ACA and LOCF, MI had the greatest proportion of negative and positive samples across all treatment weeks with minimal censoring and no missing samples.

**Fig. 2:**
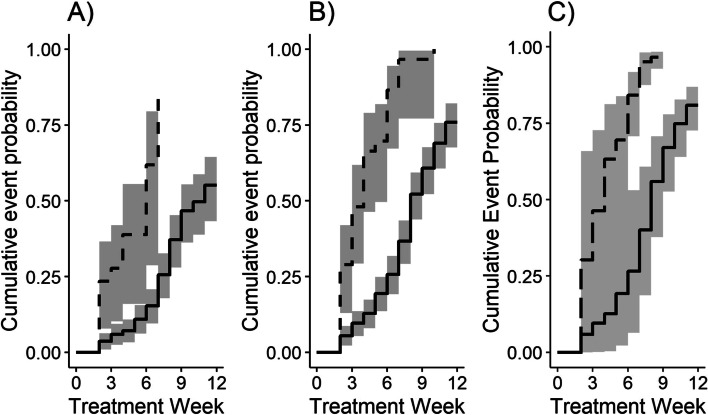
Kaplan-Meier curves of cumulative probability of culture conversion for smear negative and smear positive. Solid line is smear negative and dashed line is smear positive. Shaded regions represent 95% confidence intervals. Method for handling missing data: available case analysis **A**), last observation carried forward **B**), multiple imputation by fully conditional specification **C**)

**Fig. 3:**
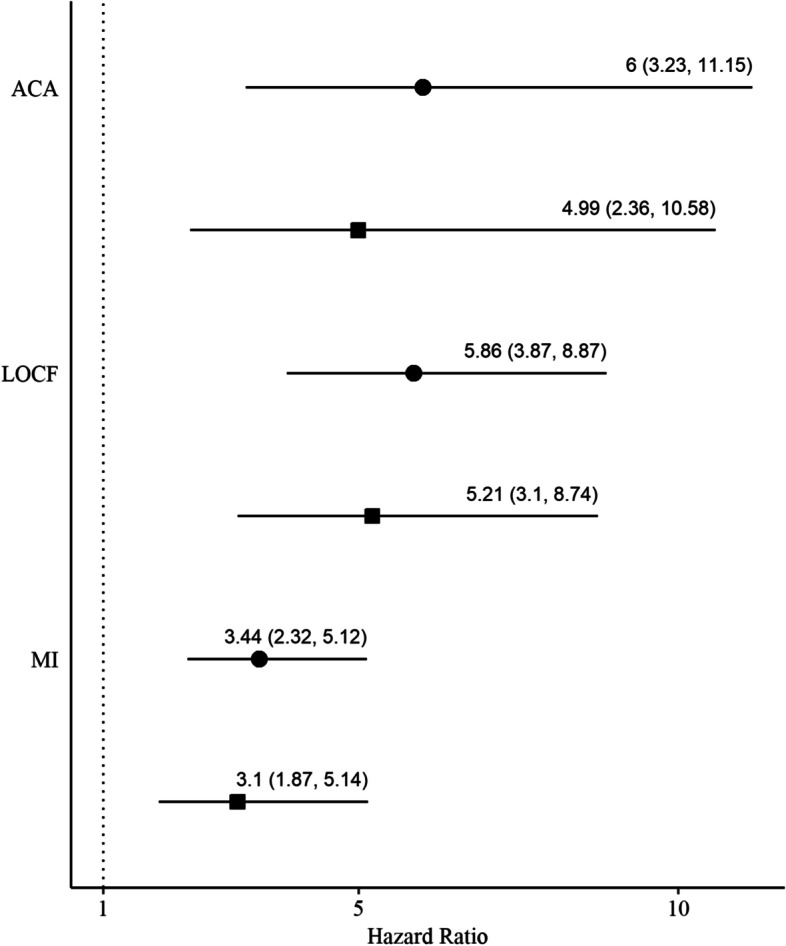
Forest plot showing hazard ratios for smear negative participants. Hazard ratios and 95% confidence intervals for negative smear with positive smear as the referent are shown for unadjusted estimates (circle) and adjusted estimates (square). Available case analysis, ACA; last observation carried forward, LOCF; multiple imputation by fully conditional specification, MI

The three missing data methods lead to considerable differences in the number of participants achieving conversion; 78 (32.8%) participants converted with ACA, 154 (64.7%) converted with LOCF, and 184 (77.1%) converted with MI. Kaplan-Meier curves were estimated stratified by smear status (Fig. 2). There was no evidence that the assumption of proportional hazards was violated (see Additional File 2). All hazard ratios for the association between smear status and culture conversion were significant and showed smear negative participants were more likely to achieve culture conversion (Fig. 3). The adjusted hazard ratio for negative smear was similar for both ACA and LOCF (HR = 4.99, 95% CI: 2.36, 10.58; HR = 5.21, 95% CI: 3.1, 8.74). The adjusted hazard ratio for MI was smallest in magnitude and had the narrowest confidence interval (HR = 3.1, 95% CI: 1.87, 5.14). RMSTD over 8 weeks was 1.48, 2.87 and 2.74 weeks for ACA, LOCF and MI, respectively.

## Discussion

**Fig. 4:**
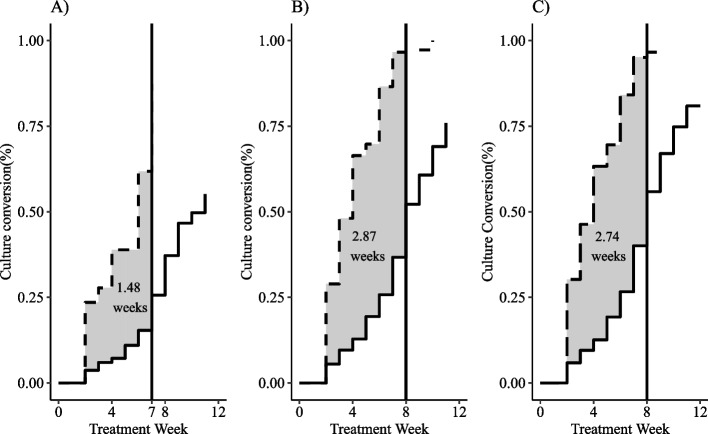
Restricted mean survival time difference for smear negative – smear positive using 8-week time horizon. Method for handling missing data: available case analysis **A**), last observation carried forward **B**), multiple imputation by fully conditional specification **C**).  Time horizon of 7 weeks was used for ACA because all smear negative participants converted or were censored by week 7. Shaded regions represent unadjusted estimates of restricted mean survival time difference

We described three different approaches to handling missing data for a time-to-event outcome dependent on serially sampling with a confirmatory result. Using data from a prospective TB cohort, we demonstrated the impact these approaches can have on estimation and inference from survival analysis methods. We estimated Kaplan-Meier curves, Cox proportional hazards models, and differences in restricted mean survival times to estimate the association between smear status and culture conversion. The number of participants achieving culture conversion with MI (184, 77.1%) was more than double the number achieving conversion with ACA (78, 32.8%). Hazard ratios for ACA and LOCF were larger with wider confidence intervals compared to MI (Fig. 3). Smear negative participants were 3.1 times more likely to achieve culture conversion compared to smear positive participants. The RMSTD over 8 weeks was about 3 weeks for LOCF and MI (Fig. 4), which was two times greater than the RMSTD for ACA. We can interpret this to mean that participants who were smear negative at baseline achieved culture conversion about 3 weeks faster on average compared with those who were smear positive at baseline. The differences in results from the three methods demonstrate the bias ad hoc methods for addressing missing data can introduce into survival analyses.

There is extensive literature on the theoretical development of multiple imputation as well as numerous tutorials with applications to different research areas [34–36]. The statistical properties and validity of multiple imputation have also been shown by many to be superior to other simple and naïve methods for addressing missing outcome and covariate data including ACA and LOCF [37]. To our knowledge, there is no prior publication that addresses the implementation of these methods with a planned survival analysis in the context of treatment for TB. Few studies have addressed missing sputum data when using culture conversion as an outcome. Scharfstein et al. addressed this problem by introducing a benchmark assumption to learn about the distribution of unknown culture results in order to estimate the overall TCC distribution [38]. Rehal compared methods for handling missing data with culture conversion handled as a binary outcome and found MI to perform best compared to ACA, LOCF, and other methods [39].

We only show empirically the differences these three methods can have on conclusions from a survival analysis. Additionally, the results shown here are subject to the analysis conducted to demonstrate these methods, most importantly the association of interest, smear status and culture conversion, and the definition of culture conversion used. There are also limitations with our imputation methods. We did not conduct a multi-level imputation to account for correlation of sputum sample results within participants. We cannot comment on the extent to which this impacts our results given the analysis we conducted. We assume our data are MAR. We showed HIV and cavitation may be associated with missingness which would indicate data MNAR but the extent to which this influences our results is unknown. Similarly, since our primary outcome is TCC, we would expect negative samples to occur more frequently in later weeks. Further analysis should be conducted to understand the impacts this has on the validity of the imputation procedures used. We do not report on or test the validity of each method or demonstrate the theoretical properties of MI. We imputed independent variable data since it was minimal in our example data set. In practice, when there is significant missing data in independent variables as well as multi-level outcome data, the imputation process becomes more complicated and great care should be taken with model specification [19]. We focus on describing the imputation of individual repeated measures as there is some evidence this performs better than imputing the composite variable, however, there may be instances where imputing the composite variable is more appropriate [40, 41].

For applications to serially collected samples for a discrete time-to-event outcome, we propose MI as the method preferred to ACA or LOCF. Our implementation of MI can be adapted to many different study designs. We described how to use MI with weekly sample collection but this can easily be extended to studies with different sampling frequencies and lengths of time between sample collection. MI is also able to impute missing covariates or outcome values, a benefit over other ad hoc methods. We chose to demonstrate these methods for a time-to-event analysis, which has more power than a simple binary outcome method of estimation. The same MI process would apply if the outcome was binary. We compared methods using the definition of two consecutive negative samples with weekly sputum collection, however, there is no standard definition of culture conversion and frequency of sample collection varies across studies [42]. The MI process detailed does not depend on the definition of culture conversion used.

TCC is often the primary outcome in phase II TB therapy trials and observational studies which both require multiple months of repeated sputum collections. These longitudinal studies are susceptible to missing specimens and it is difficult to obtain complete follow up on individuals to properly calculate and determine the true time at which culture conversion is achieved. Failing to properly address missing data can yield biased results and potentially lead to misinformed conclusions.

## Conclusions

This article addresses the common problem of missing data in longitudinal studies with repeated measures. We described two common naïve approaches and one statistically advanced approach for handling missing data. We applied these methods to data from a TB cohort and conducted a survival analysis to empirically compare methods. We showed that accounting for missing sputum data with different methods can lead to different results and conclusions. It is critical with longitudinal missing data to understand the implications and assumptions different methods for addressing missing data have and the influence this has on the final analysis. We used data from a TB study to demonstrate these concepts, however, the methods we described are broadly applicable to longitudinal missing data. Careful consideration for how to handle missing data must be taken and be pre-specified prior to analysis. We provide valuable statistical guidance and code for researchers to appropriately handle missing data in longitudinal studies.

## Supplementary Information

**Additional file 1.**

**Additional file 2.**

## Data Availability

The data that support the findings of this study are available from the corresponding author upon reasonable request. The R code used for this analysis is available at https://github.com/samalatesta/imputeTBculture.

## Abbreviations

TB: Tuberculosis
TCC: Time to culture conversion
ACA: Available case analysis
LOCF: Last observation carried forward
MI: Multiple imputation by fully conditional specification
MCAR: Missing completely at random
MAR: Missing at random
MNAR: Missing not at random
MICE: Multiple imputation by chained equations
RMSTD: Restricted mean survival time difference
TRUST: Tuberculosis Treatment and Alcohol Use Study
BMI: Body mass index
HIV: Human immunodeficiency virus
HR: Hazard ratio
CI: Confidence interval

## References

[1] World Health Organization (2021). Global tuberculosis report 2021 [internet].

[2] Rockwood N, du Bruyn E, Morris T, Wilkinson RJ (2016). Assessment of treatment response in tuberculosis. Expert Rev Respir Med.

[3] Calderwood CJ, Wilson JP, Fielding KL, Harris RC, Karat AS, Mansukhani R (2021). Dynamics of sputum conversion during effective tuberculosis treatment: a systematic review and meta-analysis. PLoS Med.

[4] Wallis RS, Doherty TM, Onyebujoh P, Vahedi M, Laang H, Olesen O (2009). Biomarkers for tuberculosis disease activity, cure, and relapse. Lancet Infect Dis.

[5] Wallis RS, Peppard T, Hermann D (2015). Month 2 culture status and treatment duration as predictors of recurrence in pulmonary tuberculosis: model validation and update. PLoS One.

[6] Phillips PPJ, Fielding K, Nunn AJ. An evaluation of culture results during treatment for tuberculosis as surrogate endpoints for treatment failure and relapse. PLoS One 2013 8(5):e63840. 10.1371/journal.pone.0063840 PMC3648512 23667677

[7] von Groote-Bidlingmaier F, Patientia R, Sanchez E, Balanag V, Ticona E, Segura P (2019). Efficacy and safety of delamanid in combination with an optimised background regimen for treatment of multidrug-resistant tuberculosis: a multicentre, randomised, double-blind, placebo-controlled, parallel group phase 3 trial. Lancet Respir Med.

[8] Conde MB, Mello FCQ, Duarte RS, Cavalcante SC, Rolla V, Dalcolmo M (2016). A phase 2 randomized trial of a Rifapentine plus moxifloxacin-based regimen for treatment of pulmonary tuberculosis. PLoS One.

[9] Lachin JM (2016). Fallacies of last observation carried forward. Clin Trials Lond Engl.

[10] Demissie S, LaValley MP, Horton NJ, Glynn RJ, Cupples LA (2003). Bias due to missing exposure data using complete-case analysis in the proportional hazards regression model. Stat Med.

[11] Hayati Rezvan P, Lee KJ, Simpson JA (2015). The rise of multiple imputation: a review of the reporting and implementation of the method in medical research. BMC Med Res Methodol.

[12] Bell ML, Fiero M, Horton NJ, Hsu CH (2014). Handling missing data in RCTs; a review of the top medical journals. BMC Med Res Methodol.

[13] Powney M, Williamson P, Kirkham J, Kolamunnage-Dona R (2014). A review of the handling of missing longitudinal outcome data in clinical trials. Trials..

[14] Salazar A, Ojeda B, Dueñas M, Fernández F, Failde I (2016). Simple generalized estimating equations (GEEs) and weighted generalized estimating equations (WGEEs) in longitudinal studies with dropouts: guidelines and implementation in R. Stat Med.

[15] Chen C, Shen B, Liu A, Wu R, Wang M (2021). A multiple robust propensity score method for longitudinal analysis with intermittent missing data. Biometrics..

[16] Little RJA, Rubin DB (2002). Statistical analysis with missing data [internet].

[17] Ross RK, Breskin A, Westreich D (2020). When is a complete-case approach to missing data valid? The importance of effect-measure modification. Am J Epidemiol.

[18] Cook RJ, Zeng L, Yi GY (2004). Marginal analysis of incomplete longitudinal binary data: a cautionary note on LOCF imputation. Biometrics..

[19] Buuren S van. Flexible imputation of missing data, 2nd Ed CRC Press; 2018. 444.

[20] Grund S, Lüdtke O, Robitzsch A (2018). Multiple imputation of missing data for multilevel models: simulations and recommendations. Organ Res Methods.

[21] Wijesuriya R, Moreno-Betancur M, Carlin JB, Lee KJ (2020). Evaluation of approaches for multiple imputation of three-level data. BMC Med Res Methodol.

[22] van Buuren S (2007). Multiple imputation of discrete and continuous data by fully conditional specification. Stat Methods Med Res.

[23] Buuren S van, Groothuis-Oudshoorn K. Mice: multivariate imputation by chained equations in R. J Stat Softw 2011 45:1–67.

[24] Huque MH, Carlin JB, Simpson JA, Lee KJ (2018). A comparison of multiple imputation methods for missing data in longitudinal studies. BMC Med Res Methodol.

[25] White IR, Royston P, Wood AM (2011). Multiple imputation using chained equations: issues and guidance for practice. Stat Med.

[26] Rubin DB (2004). Multiple imputation for nonresponse in surveys.

[27] Royston P, Parmar MK (2013). Restricted mean survival time: an alternative to the hazard ratio for the design and analysis of randomized trials with a time-to-event outcome. BMC Med Res Methodol.

[28] Uno H, Claggett B, Tian L, Inoue E, Gallo P, Miyata T (2014). Moving beyond the Hazard ratio in quantifying the between-group difference in survival analysis. J Clin Oncol.

[29] Marshall A, Altman DG, Holder RL, Royston P (2009). Combining estimates of interest in prognostic modelling studies after multiple imputation: current practice and guidelines. BMC Med Res Methodol.

[30] Morisot A, Bessaoud F, Landais P, Rébillard X, Trétarre B, Daurès JP (2015). Prostate cancer: net survival and cause-specific survival rates after multiple imputation. BMC Med Res Methodol.

[31] Myers B, Bouton TC, Ragan EJ, White LF, McIlleron H, Theron D (2018). Impact of alcohol consumption on tuberculosis treatment outcomes: a prospective longitudinal cohort study protocol. BMC Infect Dis.

[32] van Zyl-Smit RN, Binder A, Meldau R, Mishra H, Semple PL, Theron G (2011). Comparison of quantitative techniques including Xpert MTB/RIF to evaluate mycobacterial burden. PLoS One.

[33] Tian L, Zhao L, Wei LJ (2014). Predicting the restricted mean event time with the subject’s baseline covariates in survival analysis. Biostat Oxf Engl.

[34] Austin PC, White IR, Lee DS, van Buuren S (2021). Missing data in clinical research: a tutorial on multiple imputation. Can J Cardiol.

[35] Kropko J, Goodrich B, Gelman A, Hill J (2014). Multiple imputation for continuous and categorical data: comparing joint multivariate Normal and conditional approaches. Polit Anal.

[36] Tan PT, Cro S, Van Vogt E, Szigeti M, Cornelius VR (2021). A review of the use of controlled multiple imputation in randomised controlled trials with missing outcome data. BMC Med Res Methodol.

[37] White IR, Carlin JB (2010). Bias and efficiency of multiple imputation compared with complete-case analysis for missing covariate values. Stat Med.

[38] Scharfstein D, Rotnitzky A, Abraham M, McDermott A, Chaisson R, Geiter L (2015). On the analysis of tuberculosis studies with intermittent missing sputum data. Ann Appl Stat.

[39] Rehal S (2018). Implications of missing data in tuberculosis non-inferiority clinical trials.

[40] Pan Y, He Y, Song R, Wang G, An Q (2020). A passive and inclusive strategy to impute missing values of a composite categorical variable with an application to determine HIV transmission categories. Ann Epidemiol.

[41] Nguyen CD, Carlin JB, Lee KJ (2021). Practical strategies for handling breakdown of multiple imputation procedures. Emerg Themes Epidemiol.

[42] Weir IR, Wasserman S (2021). Treatment effect measures for culture conversion endpoints in phase IIb tuberculosis treatment trials. Clin Infect Dis.

