# Supplementary material for: Methods for handling missing data in serially sampled sputum specimens for mycobacterial culture conversion calculation
Source: BMC Med Res Methodol. 2022 Nov 19;22:297. doi: 10.1186/s12874-022-01782-8 (PMC9675206; doi:10.1186/s12874-022-01782-8)
Supplement: Supplementary file 1 — Additional file 1. [file 12874_2022_1782_MOESM1_ESM.docx]

Additional File 1

Table 1. Non-missing values for independent variables used in analysis

| Independent Variable | Overall  N=261 |
| --- | --- |
| Biologic Sex | 261 (100.0%) |
| Age | 261 (100.0%) |
| BMI | 261 (100.0%) |
| HIV Status | 260 (99.6%) |
| Excessive Alcohol Use | 261 (100.0%) |
| Tobacco Use | 261 (100.0%) |
| Cavitation | 245 (93.9%) |
| Smoked Substance Use | 261 (100.0%) |
