# Supplementary material for: Methods for handling missing data in serially sampled sputum specimens for mycobacterial culture conversion calculation
Source: BMC Med Res Methodol. 2022 Nov 19;22:297. doi: 10.1186/s12874-022-01782-8 (PMC9675206; doi:10.1186/s12874-022-01782-8)

Additional File 2


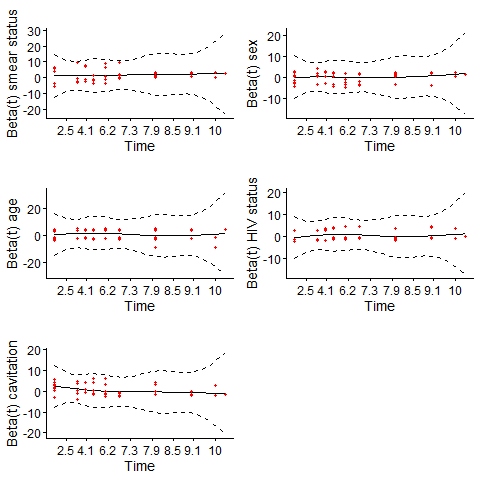
Figure 1. Schoenfeld residual plots for each predictor included in cox proportional hazards models. Method for handling missing data: available case analysis A), last observation carried forward B), multiple imputation by fully conditional specification C). Only residuals for first imputed data set for MI are shown.

A)

B)


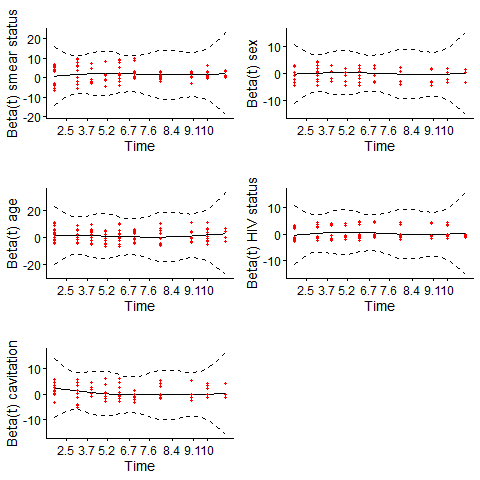


C)


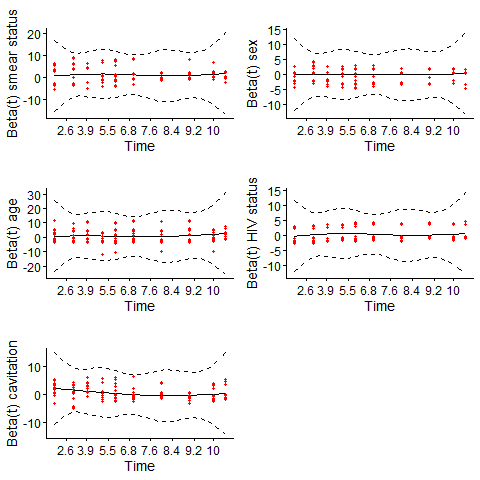

Supplement: Supplementary file 2 — Additional file 2. [file 12874_2022_1782_MOESM2_ESM.docx]
